# Assembly and analysis of the mitochondrial genome of *Prunella vulgaris*

**DOI:** 10.3389/fpls.2023.1237822

**Published:** 2023-08-02

**Authors:** Zhihao Sun, Ya Wu, Pengyu Fan, Dengli Guo, Sanyin Zhang, Chi Song

**Affiliations:** ^1^ Institute of Herbgenomics, Chengdu University of Traditional Chinese Medicine, Chengdu, China; ^2^ Wuhan Benagen Technology Co., Ltd, Wuhan, Hubei, China; ^3^ Innovative Institute of Chinese Medicine and Pharmacy, Chengdu University of Traditional Chinese Medicine, Chengdu, China

**Keywords:** *Prunella vulgaris*, mitochondrial genome, codon usage, repeated sequence, evolution

## Abstract

*Prunella vulgaris* (Lamiaceae) is widely distributed in Eurasia. Former studies have demonstrated that *P. vulgaris* has a wide range of pharmacological effects. Nevertheless, no complete *P. vulgaris* mitochondrial genome has been reported, which limits further understanding of the biology of *P. vulgaris*. Here, we assembled the first complete mitochondrial genome of *P. vulgaris* using a hybrid assembly strategy based on sequencing data from both Nanopore and Illumina platforms. Then, the mitochondrial genome of *P. vulgaris* was analyzed comprehensively in terms of gene content, codon preference, intercellular gene transfer, phylogeny, and RNA editing. The mitochondrial genome of *P. vulgaris* has two circular structures. It has a total length of 297, 777 bp, a GC content of 43.92%, and 29 unique protein-coding genes (PCGs). There are 76 simple sequence repeats (SSRs) in the mitochondrial genome, of which tetrameric accounts for a large percentage (43.4%). A comparative analysis between the mitochondrial and chloroplast genomes revealed that 36 homologous fragments exist in them, with a total length of 28, 895 bp. The phylogenetic analysis showed that *P. vulgaris* belongs to the Lamiales family Lamiaceae and *P. vulgaris* is closely related to *Salvia miltiorrhiza*. In addition, the mitochondrial genome sequences of seven species of Lamiaceae are unconservative in their alignments and undergo frequent genome reorganization. This work reports for the first time the complete mitochondrial genome of *P. vulgaris*, which provides useful genetic information for further *Prunella* studies.

## 1 Introduction

*P. vulgaris* is a low-growing herbaceous perennial widely distributed in Eurasia’s temperate and tropical mountainous regions. The mature spikes of *P. vulgaris* are cylindrical and slightly flat. Its stems are relatively short. The panicle consists of several whorls of persistent calyxes and bracts, ranging from a few to ten. In southeastern China, the fresh leaves of *P. vulgaris* are served as a vegetable (Bai et al., 2016). The spikes of dried fruits of *P. vulgaris* are considered to have anti-inflammatory action in traditional Chinese medicine (Li et al., 2015; Liu et al., 2020). Modern pharmacological studies have revealed the presence of natural compounds with anti-inflammatory, antibacterial, antioxidant, and immunomodulatory properties in *P. vulgaris*, e.g., triterpenes, phenolic acid, and oleanolic acid (Bai et al., 2016). These compounds are important for pharmaceutical research (Su et al., 2022). The study of *P. vulgaris* organelle genome is essential to better exploit its medicinal and economic value. Important plastid organelles, i.e., mitochondria and chloroplast, play key roles in plant development and reproduction, and their contributions to energy metabolism and material conversion depend on their own semi-autonomous genetic systems (Nielsen et al., 2010; Gualberto et al., 2014). Besides, compared with whole genome assembly, the assembly of plastid genome is more cost-effective and could provide useful information for the evolutionary analysis of the focal species. The complete chloroplast genome of *P. vulgaris* has already been assembled (Han and Zheng, 2018). The present study reveals the complete mitochondrial genome of *P. vulgaris*, providing the necessary genetic sequence for further phylogeny and resource utilization.

As an important component of most eukaryotic cells, mitochondria have energy conversion, biosynthetic, and signaling functions. Mitochondria can encode some proteins semi-autonomously, but these processes are regulated by nuclear-encoded genes (Mackenzie and McIntosh, 1999). Thus, abnormal mitochondrial gene expression in *Brassica napus* may lead to male sterility(Liu et al., 2017). The characteristics of plant mitochondrial genomes include the existence of highly conserved genes, a large number of genomic structural rearrangements, a wide range of non-coding sequences, and extensive RNA editing (Silvestris et al., 2020). Mitochondrial genomes usually exhibit matrilineal inheritance, which provides useful information about evolution and phylogeny of the focal species (Birky, 2001). For example, the mitochondrial genome sequence of *Brassica oleracea* facilitated the evolutionary analysis of this species(Shao et al., 2021). The structure of plant mitochondrial genomes may be linear or multi-branched (Wang et al., 2019; Jackman et al., 2020). The reasons for the structural diversity of plant mitochondrial genomes are still unclear. The transfer of DNA between the mitochondrial genome and the chloroplast genome is a common event in the plant genome. Some studies believe that this event usually leads to changes in the length of mitochondrial genome and changes the structure of mitochondrial genome (Allen, 2015; Turmel et al., 2016). With the rapid development of genome assembly and sequencing technologies, complete organelle genomes of plants are able to be assembled. This will facilitate our understanding of plants, for example, by detecting nucleotide fragments of gene insertions or deletions at the same position in different mitochondrial genomes to distinguish species (Chen et al., 2022).

In this study, we assembled the first complete *P. vulgaris* mitochondrial genome using a hybrid assembly strategy based on sequencing data from Illumina and Nanopore. The assembled mitochondrial genome was annotated from Illumina and Nanopore platforms. The characteristic information of the mitochondrial genome of *P. vulgaris* was discussed in terms of codon usage preference, genome repeat sequence, and genes transfer between the mitochondrial genome and chloroplast genome. Phylogenetic tree and synteny analysis provide hints about the evolutionary history of *P. vulgaris*. The results of this study will provide useful information for the mitochondrial genome of *P. vulgaris*.

## 2 Materials and methods

### 2.1 *P. vulgaris* DNA extraction and mitochondrial genome assembly

The *P. vulgaris* plants were collected from wild in Hubei province, China, and cultured in Wuhan, China (31°68’ N, 118°45’ E). High quality genomic DNA were isolated from fresh leaves using the standard CTAB method (Arseneau et al., 2017; Cheng et al., 2021). Illumina and Nanopore platforms were used for sequencing. Illumina sequencing and Oxford sequencing were performed by Wuhan Benagen Tech Solutions Company (http://en.benagen.com/). Illumina sequencing data was sequenced using the HiSeq Xten PE150 Illumina, San Diego, CA, USA sequencing platform and Nanopore sequencing was performed by Oxford Nanopore GridION × 5 Oxford Nanopore Technologies, Oxford, UK. GetOrganelle (v1.7.5) (Jin et al., 2020) was used to perform plant mitochondrial genome assembly (default parameters) and a graphical plant mitochondrial genome was obtained. Since the graphical genome generated by GetOrganelle comprised multiple nodes, with redundant fragments existing in the border of two neighbor nodes, Bandage (Wick et al., 2015) was used to visualize the graphical genome and Nanopore data was mapped to help manually check these redundant fragments. BWA (0.7.17) is used to map the third generation sequencing data to the graphical genome, followed by manually identification and removing of the redundant fragments (Li and Durbin, 2009).

### 2.2 Annotation of the mitochondrial genome of *P. vulgaris*

The *P. vulgaris* mitochondrial genomes were annotated using Geseq (Tillich et al., 2017) with Arabidopsis thaliana (Sloan et al., 2018) and Liriodendron tulipifera (Richardson et al., 2013) as reference genomes. The *P. vulgaris* mitochondrial genomes were annotated using Geseq (Tillich et al., 2017). The tRNA genes were annotated using the tRNAscan-SE (Lowe and Eddy, 1997). The rRNA genes were annotated using BLASTN (Chen et al., 2015). Each mitochondrial genome annotation error was manually corrected using Apollo (Lewis et al., 2002).

### 2.3 Relative synonymous codon usage

The protein coding sequences of the genome were extracted using Phylosuite (Zhang et al., 2020). Codon preferences of protein-coding genes in the mitochondrial genome were analyzed by Mega (v7.0). The results of the analysis are expressed in relative synonymous codon usage (RSCU).

### 2.4 Analysis of repeated sequences

The online version of MISA (Beier et al., 2017) was used to analyze Simple Sequence Repeat (SSR) in assembled mitochondrial genome. To define the SSR locus, we searched that SSRs with a length of 1, 2, 3, 4, 5, and 6 bases have at least 10, 5, 4, 3, 3, and 3 repeats, respectively. The online version of TRF (Benson, 1999) was used to identify tandem repeat sequences in the mitochondrial genome, with alignment parameters: match = 2, mismatch = 5, and indels = 7. REPuter web server (Kurtz et al., 2001) was used to identify dispersed repeats with the following options: maximum computed repeats = 5000, hamming distance = 3, and minimal repeat size = 30.

### 2.5 Homologous fragments between chloroplast genome and mitochondrial genome

The chloroplast genome of *P. vulgaris* was reassembled by GetOrganelle (Jin et al., 2020) based on the Illumina and Nanopore sequencing data and then annotated by CPGAVAS2 (Shi et al., 2019). The assembled chloroplast genome size is 151, 346 bp (**Figure S1**). Identification of homologous fragments between chloroplast genome and mitochondria genome of *P. vulgaris* using the BLASTN online tool on the NCBI website (https://www.ncbi.nlm.nih.gov/), with the default parameters. Results with an identity value greater than 75 were retained (**Table S1**). The results were visualized using the RCircos package (Zhang et al., 2013).

### 2.6 Phylogenetic tree construction and synteny analysis

A phylogenetic tree was constructed for 22 species (**Table S2**) from 6 families of Lamiales based on the DNA sequences of 16 conserved mitochondrial PCGs (*atp1, atp4, ccmB, ccmC, ccmFC, ccmFN, cob, cox2, cox3, matR, nad1, nad2, nad3, nad5, nad6, rps13*). PhyloSuite (Zhang et al., 2020) and MAFFT(Katoh and Standley, 2013) were used to extract shared genes and align multiple sequences. IQ-TREE (Nguyen et al., 2015) was used to build the phylogenetic tree. ModelFinder (Kalyaanamoorthy et al., 2017; Zhang et al., 2020) was used to find the most suitable model from for out data based on Akaike information criterion (AIC). The ‘TVM+F+I+G4’ model was finally chosen for maximum likelihood tree construction. iTOL (https://itol.embl.de/) (Letunic and Bork, 2019) was used to visualize the results of phylogenetic analysis. The mitochondrial genomes of seven Lamiaceae species (*Scutellaria barbata, Pogostemon heyneanus, Salvia miltiorrhiza, P. vulgaris, Ajuga reptants, Rotheca serrata*, and *Vitex trifolia*) were compared using the BLAST program. Then homologous sequences longer than 500 bp were retained as conserved collinearity blocks.

### 2.7 RNA editing event analysis methods

Prediction of RNA editing events was performed by the online version of PREPACT3 (http://www.prepact.de/) (Lenz et al., 2018). RNA editing sites of a total of 29 unique PCGs were predicted with a cutoff value of 0.001.

## 3 Results

### 3.1 Genomic features of the *P. vulgaris* mitochondrial genome

**Figure 1:**
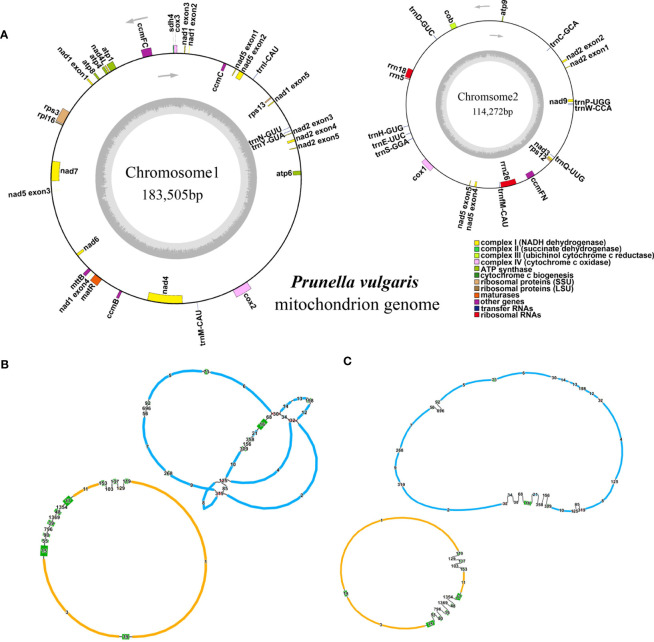
The mitochondrial genome structure and annotation of *P. vulgaris*. **(A)** Annotations of *P. vulgaris* mitochondrial genome. **(B)** Two circular contigs of *P. vulgaris* mitochondrial genome predicted by GetOrganelle. **(C)** The 2D structure of *P. vulgaris* mitochondrial genome after removing artificial chloroplast and nuclear gene fragments. In B and C, the red nodes represent the predicted duplication regions and the green nodes represent the predicted segments migrating to the mitochondrial genomes from chloroplast.

In this study, the *P. vulgaris* mitochondrial genome was assembled. It is composed of two circular structures (**Figure 1A**). We used Bandage (Wick et al., 2015) to visualize the mitochondrial genome assembled based on Illumina data. Duplicated regions were removed with the help of the third-generation sequencing reads. Manual method was used to remove the nodes formed by nuclear and chloroplast genes. The assembled raw mitochondrial genome contains 47 nodes that including predicted duplication regions and mitochondrial genomic regions migrating from chloroplast (**Figure 1B**). After manually removing of redundant fragments, two clear circular contigs were obtained (**Figure 1C**). The total length of the assembled genome was 297, 777 bp and the GC content was 43.92% (**Table 1**). The mitochondrial genome of *P. vulgaris* was annotated with 29 unique PCGs (**Figure 1A**), 13 tRNA genes and 3 rRNA genes (**Table 2**). The unique PCGs include five ATP synthase genes (*atp1, atp4, atp6, atp8* and *atp9*), nine NADH dehydrogenase genes (*nad1, nad2, nad3, nad4, nad4L, nad5, nad6, nad7* and *nad9*), four ubiquinol cytochrome c reductase genes (*ccmB, ccmC, ccmFC* and *ccmFN*), three cytochrome c oxidase genes (*cox1, cox2* and *cox3*), one transport membrane protein gene (*mttB*), one maturases gene (*matR*), one cytochrome c biogenesis gene (*cob*), one large subunit of ribosome gene (*rpl16*), three small subunit of ribosome genes (*rps3, rps12* and *rps13*), and one succinate dehydrogenase gene (*sdh4*).

**Table 1:** Information on the mitochondrial genome of *P. vulgaris*.

Contigs	Type	Length	GC content
Chromosome 1-2	Branched	297, 777 bp	43.92%
Chromosome 1	Circular	183, 505 bp	44.27%
Chromosome 2	Circular	114, 272 bp	43.36%

**Table 2:** The encoding genes of *P. vulgaris* mitochondrial genome.

Group of genes	Name of genes
ATP synthase	*atp1*, *atp4*, *atp6*, *atp8*, *atp9*
NADH dehydrogenase	*nad1*, *nad2*, *nad3*, *nad4*, *nad4L*, *nad5*, *nad6*, *nad7*, *nad9*
Cytochrome c biogenesis	*cob*
Ubiquinol cytochrome c reductase	*ccmB*, *ccmC*, *ccmFC*, *ccmFN*
Cytochrome c oxidase	*cox1*, *cox2*, *cox3*
Maturases	*matR*
Transport membrane protein	*mttB*
Large subunit of ribosome	*rpl16*
Small subunit of ribosome	*rps3*, *rps12*, *rps13*
Succinate dehydrogenase	*sdh4*
Ribosome RNA	*rrn5*, *rrn18*, *rrn26*
Transfer RNA	*trnC-GCA*, *trnD-GUC*, *trnE-UUC*, *trnfM-CAU*, *trnH-GUG*, *trnI-CAU*, *trnM-CAU*, *trnN-GUU*, *trnP-UGG*, *trnQ-UUG*, *trnS-GGA*, *trnW-CCA*, *trnY-GUA*

### 3.2 Codon usage analysis of PCGs

**Figure 2:**
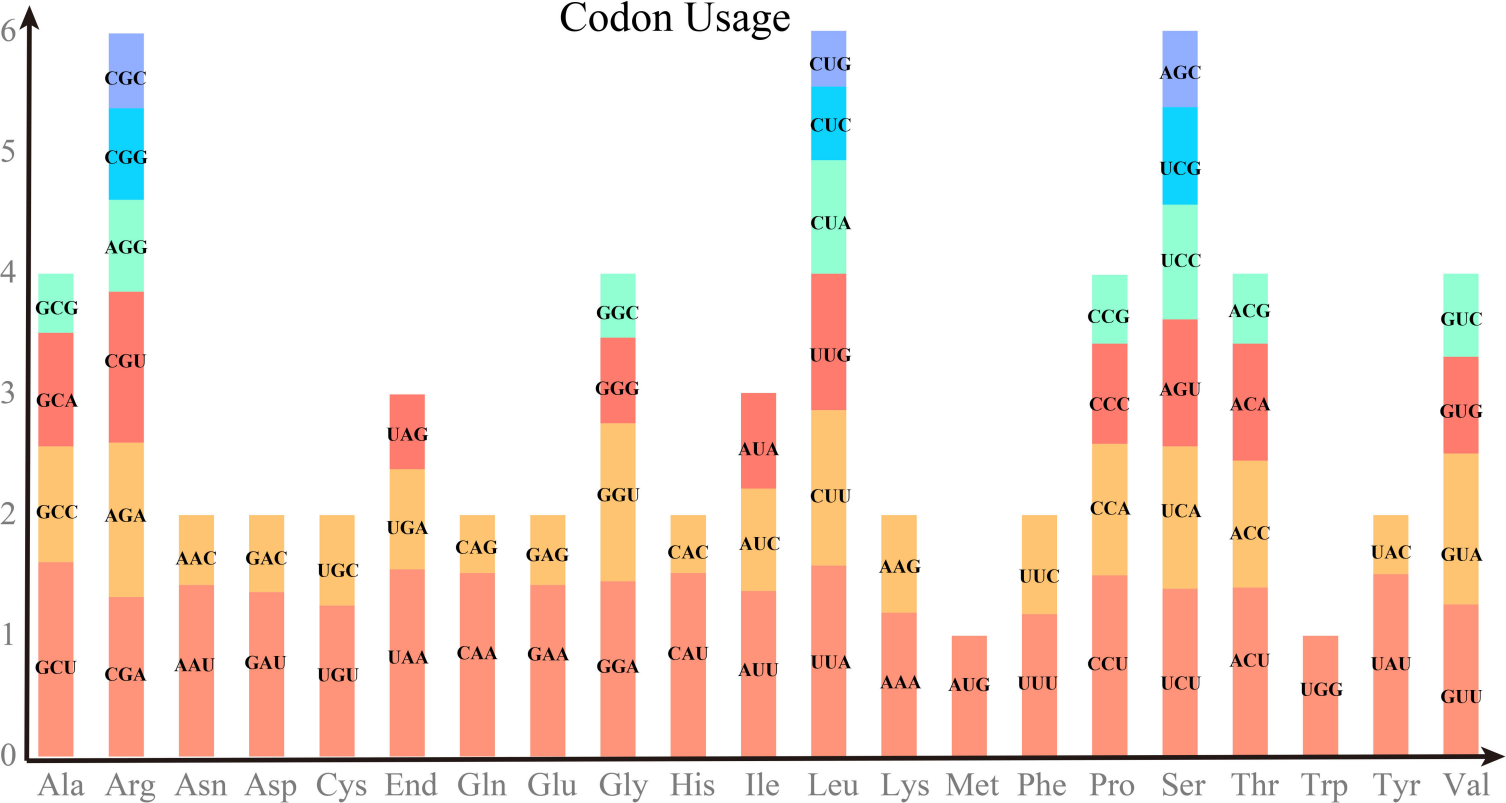
RSCU values of PCGs on *P. vulgaris* mitochondrial genome. The horizontal coordinate represents the 20 amino acids and the end codon. Vertical coordinates indicate the frequency of use. Different codons of the same amino acid are colored differently.

Codon preference analysis was performed on 29 unique PCGs of *P. vulgaris* mitochondria. The codon usage by individual amino acids is shown in **Table S3**. Relative synonymous codon usage (RSCU) value greater than 1 indicated that the corresponding amino acid was preferentially used. As shown in **Figure 2**, the Methionine (Met) codon AUG and Tryptophan (Try) code UGG, which both have the RSCU value of 1. There is also a general preference for codon use in the PCGs of the mitochondria. For example, Alanine (Ala) has a high preference for GCU with the highest RSCU value of 1.61 among mitochondrial PCGs, followed by Leucine (Leu) with a usage preference for UUA. Notably, the maximum RSCU values of Lysine (Lys) and Phenylalanine (Phe) were less than 1.2 and did not have a strong codon usage preference.

### 3.3 Repeated sequence analysis of the mitochondrial genome of *P. vulgaris*

**Figure 3:**
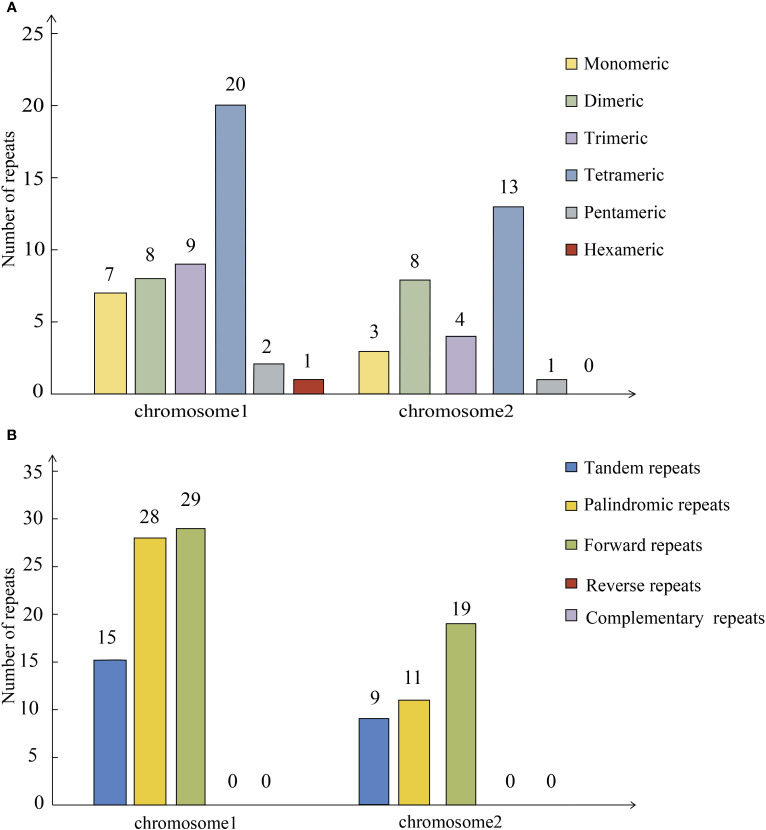
Horizontal coordinate indicates mitochondrial molecules and vertical coordinate indicates the number of repeat fragments. **(A)** Simple Sequence Repeat of *P. vulgaris* mitochondrial genome. **(B)** Repeated sequence of *P. vulgaris* mitochondrial genome.

In MISA online prediction for the chromosome 1, a hit was retained as a SSR when it met two criteria: the match score should be greater than 69% and length should be between 10 and 33 bp. A total of 47 SSRs were found on mitochondrial chromosome 1, with monomeric and dimeric forms accounting for 31.91% of the total SSRs. Adenine and thymine monomeric repeats accounted for 85.71% (6) of the 7 monomeric SSRs. There is a hexameric SSRs in chromosome 1 (**Figure 3A**). Tandem repeats are widely found in eukaryotic and prokaryotes genomes. Mitochondrial chromosome 1 contains 15 tandem repeats. Repetitive dispersed sequences in mitochondrial chromosome 1 were examined. A total of 57 pairs of repetitive sequences with lengths greater than or equal to 30 bp were observed (**Figure 3B**). Among them, 28 pairs of palindromic repeats and 29 pairs of forward repeats were detected. The length of the longest palindromic repeat was 1, 392 bp and that of the longest forward repeat was 1, 429 bp.

In MISA online prediction for the chromosome 2, a hit was retained as a SSR when it met two criteria: the match score should be greater than 71% and length should be between 9 and 33 bp. A total of 29 SSRs were found in mitochondrial chromosome 2, with monomeric and dimeric forms accounting for 37.93% of total SSRs. Adenine and thymine monomeric repeats accounted for 66.67% of the three monomeric SSRs. Mitochondrial chromosome 2 contains nine tandem repeats. A total of 30 pairs of repetitive sequences with lengths greater than or equal to 30 bp were observed (**Figure 3B**). Among them, 11 pairs of palindromic repeats and 19 pairs of forward repeats were detected. The length of the longest palindromic repeat was 54 bp and that of longest forward repeat was 51 bp.

### 3.4 DNA migration from chloroplast to mitochondria

**Figure 4:**
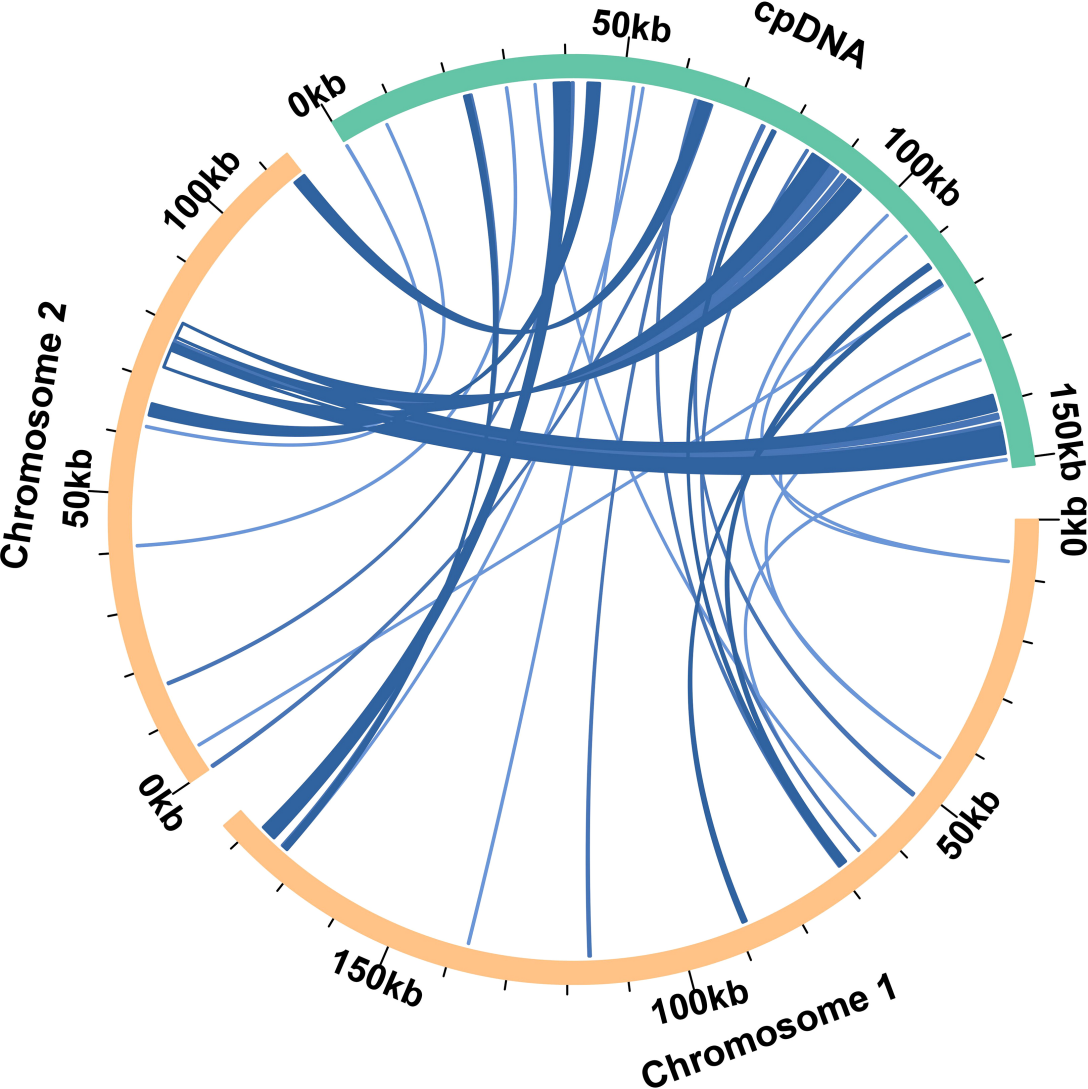
The brown arc in the figure represents the mitochondrial genome, the green arc represents the chloroplast genome. The genome fragments corresponding to the blue connecting lines between arcs are homologous fragments.

Based on the analysis of sequence similarity, a total of 36 fragments were homologous between the mitochondrial and chloroplast genomes (**Figure 4**). The total length of these homologous fragments was 28, 895 bp, accounting for 9.70% of the total length of the mitochondrial genome. The longest fragments were fragment 19 and fragment 20, both of which were 3, 276 bp (**Table S1**). By annotating these homologous sequences, 16 complete genes were identified on 36 homologous fragments (**Table S1**), including 10 PCGs (*ndhB, ndhI, psbJ, psbL, psbF, psbE, petL, petG, rps4*, and *ycf15*) and six tRNA genes (*trnD-GUC, trnH-GUG, trnM-CAU, trnP-UGG, trnS-GGA*, and *trnW-CCA*).

### 3.5 Phylogenetic analysis and synteny analysis based on mitochondrial genomes of higher plants

**Figure 5:**
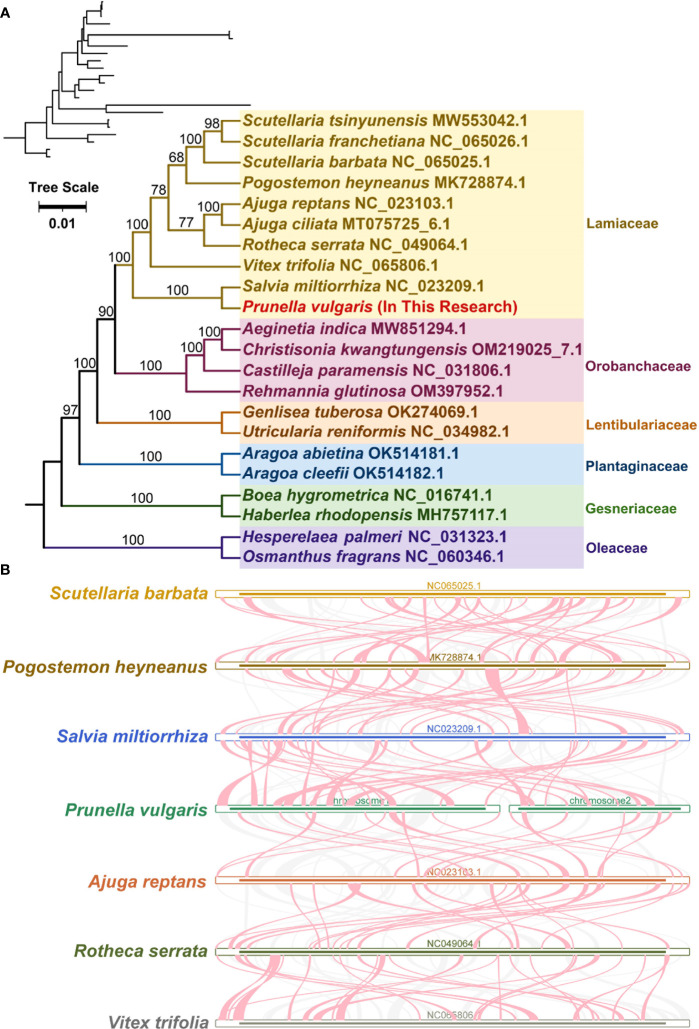
Evolution analysis of *P. vulgaris*. **(A)** The plants in the diagram belong to of Lamiales. Different families are represented by different colors, with *P. vulgaris* represented in red. **(B)** Red-curved regions indicate where inversions occur, gray regions indicate regions of good homology, and white regions indicate species-unique sequences.

A phylogenetic analysis was performed with 22 species based on the DNA sequences of 16 conserved mitochondrial PCGs (*atp1, atp4, ccmB, ccmC, ccmFC, ccmFN, cob, cox2, cox3, matR, nad1, nad2, nad3, nad5, nad6*, and *rps13)*.The two mitochondrial genomes of Oleaceae were set as outgroups (**Figure 5A**). The results showed that *P. vulgaris* belongs to the Lamiales family Lamiaceae and is closely related to *Salvia miltiorrhiza*. The topology of this mitochondrial DNA-based phylogeny is consistent with the Angiosperm Phylogeny Group IV (Bennett and Alarcón, 2015).

A large number of homologous collinearity blocks were detected in Lamiaceae species (**Figure 5B**). No collinearity blocks with lengths less than 0.5 kb were retained. In addition, some regions were unique to *P. vulgaris*, i.e., have no homologous region with any other species. The collinearity blocks are not in the same order. The mitochondrial genome sequences of these seven Lamiaceae species are not sequentially conserved and have undergone frequent genomic rearrangements.

### 3.6 The prediction of RNA editing events

**Figure 6:**
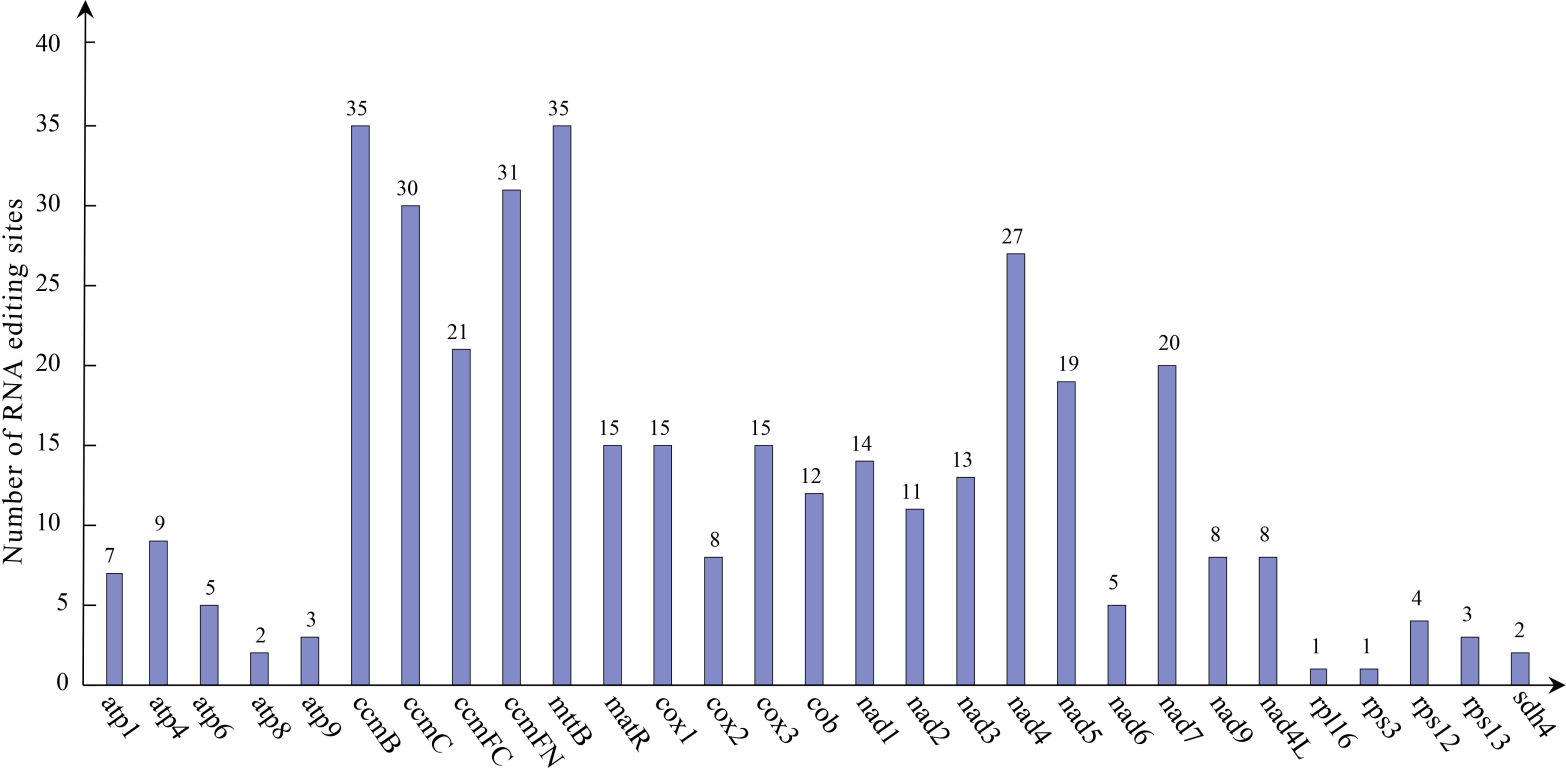
Predicted RNA editing events in *P. vulgaris* mitochondrial genes. Horizontal coordinate indicates represents different genes and vertical coordinate indicates the predicted number of RNA editing events.

A total of 379 potential RNA editing sites were identified on 29 mitochondrial PCGs (**Figure 6**), which are dominantly base C to U editing (**Table S1**). Both *ccmB* and *mttB* had the highest number of edits among all mitochondrial genes (35 RNA editing sites identified), followed by *ccmFN* with 31 RNA editing events. In addition, *rpl16* and *rps3* had the lowest number of edits among all mitochondrial genes (one RNA editing sites identified).

## 4 Discussion

In general, plants contain three genomes, the nuclear genome, the plastid genome and the mitochondrial genome. Recent advances in plant whole genome study such as genome assembly or single cell sequencing have greatly facilitated medicinal plant research; however, plastid genome remains to be a powerful and cost-effective way(Guo et al., 2022; Chen et al., 2023; Sun et al., 2023). The *P. vulgaris* chloroplast genome has been sequenced (Han and Zheng, 2018), but no mitochondrial sequencing has been completed for this species. In this study, the *P. vulgaris* mitochondrial genome was assembled into two circular structures with a total length of 297, 777 bp. Due to the simplicity of codons, each amino acid corresponds to at least 1 codon, and there are up to six corresponding codons. The codons of one amino acid are often used at different frequencies. Codons may correlate with gene expression levels (Trotta, 2013; Hia and Takeuchi, 2021). The use of genetic codons varies greatly from species to species, which provides additional information on species-specific evolution. Gene expression levels and gene length, tRNA abundance and interactions, and codon position in the gene are some of the factors that influence codon preference. There is a clear codon usage preference in *P. vulgaris* mitochondria, with differences in the frequency of different codons of each amino acid being used, except for Met and Trp. For example, AAU and AAC are synonymous codons of Asn, in which AAU was used 71% and AAC was used 29%. Codon usage preference has been used to study phylogeny and molecular evolution of genes among organisms. By studying codon preference in *Brassica campestris* was found that selection pressure plays most of the role in mutational pressure(Paul et al., 2018; Parvathy et al., 2022). In addition, codon usage preference should be considered when designing high yield and resistance genes.

Tandem repeat sequences are one of the most prevalent features of genomic sequences. Tandem repeat sequences have important roles in biological evolution, gene regulation, gene expression, and genome stability. A total of 58 SSRs were found in the mitochondria of *P. vulgaris*, which provided great convenience for genetic studies and species identification due to the maternal inheritance characteristics of mitochondria. SSRs have been used to classify different species, which is beneficial for species identification and breeding of superior varieties. Among tandem repeats, SSR is a special kind of tandem repeat sequence, which generally less than 6 bp. SSRs are often used for molecular marker of development due to their characteristics such as dominant inheritance. They are independent of the external environment and growth conditions (Song et al., 2015). In addition, these markers have advantages such as large numbers, stable traits, simple operation, and rapid detection, and are widely used in the analysis and identification of herbal plants (Chen et al., 2022). The mitochondrial genomes of plants and animals have formed different evolutionary features.

In general, the mutation rate of plant mitochondrial genome is lower than that of animal mitochondrial genome (Darracq et al., 2011). Plant mitochondrial genome can integrate exogenous DNA by migrating fragments with chloroplast genome (Law et al., 2022). The presence of fragments of chloroplast genes was found in the assembled mitochondrial genome of *P. vulgaris*. Mitochondrial and chloroplast gene migration is an important mechanism for biological evolution and diversity formation, which is important for evolution, adaptation, and diversity of organisms (Xiong et al., 2008). And this results in many structural variations in the plant mitochondrial genome.

Phylogenetic relationships among species are the basis for many biological studies. An accurate phylogenetic tree supports our understanding of key transitions in evolution (Kapli et al., 2020). Based on the phylogenetic tree constructed from 16 genes of mitochondria, *P. vulgaris* was more closely related to *Salvia miltiorrhiza* than other 20 species in this study. The tree matches the latest classification of the Angiosperm Phylogeny Group IV (Bennett and Alarcón, 2015). Collinearity research is a method to analyze the relationship between homologous genes or sequences. The collinearity of genes in plant genome usually decreases with the increase of evolutionary distance (Wicker et al., 2010). A large number of homologous collinearity blocks were detected in the *P. vulgaris* with the rest of Lamiales species, but these collinearity blocks were short in length. In addition, some blank regions were found. These sequences are unique to the species and have no homology with the rest of the species. The collinearity blocks were not in the same order among the mitochondrial genomes of Lamiaceae. The results indicate that the mitochondrial genome sequences of these seven Lamiaceae species are not conservative in their alignments and undergo frequent reorganization. RNA editing is the phenomenon of base insertion, deletion, or conversion that occurs in the coding region of post-transcriptional RNA, such as 441 C-to-U editing sites have been identified in *Arabidopsis thaliana* and 225 C-to-U editing sites in *Salvia miltiorrhiza*. Due to the lack of suitable transcriptome data, *P. vulgaris* was predicted through the website that it has 379 RNA editing sites, all of which are C-to-U (Yang et al., 2022).

## 5 Conclusion

In this study, we have assembled the first complete mitochondrial genome of *P. vulgaris*. It has a total length of 297, 777 bp, a GC content of 43.92%, and 29 unique PCGs. We found 76 SSRs in the mitochondrial genome. The phylogenetic analysis showed that *P. vulgaris* is closely related to *Salvia miltiorrhiza*, consistent with the Angiosperm Phylogeny Group IV. The complete mitochondrial genome of *P. vulgaris* is useful to understanding Lamiales evolution and could benefit following works such as breeding of varieties of *P. vulgaris.*

## Data availability statement

The original mitochondrial genome presented in the study are publicly available. This data can be found in NCBI (https://www.ncbi.nlm.nih.gov/) under the GenBank: OR113011.1 (https://www.ncbi.nlm.nih.gov/nuccore/OR113011.1/). The data are publicly available. The datasets presented in this study can be found in NCBI. The names of the repositories and accession numbers can be found in the **Supplementary Material**.

## Author contributions

CS conceived the study. YW and SZ collected the data. PF and DG analyzed the data. ZS wrote the manuscript. All authors contributed to the article and approved the submitted version.
