# Supplementary material for: Assembly and analysis of the mitochondrial genome of Prunella vulgaris
Source: Front Plant Sci. 2023 Aug 2;14:1237822. doi: 10.3389/fpls.2023.1237822 (PMC10433383; doi:10.3389/fpls.2023.1237822)
Supplement: Supplementary file 1 [file DataSheet_1.docx]

## Figure S1. Reassembled *Prunella vulgaris* chloroplast genome.

**
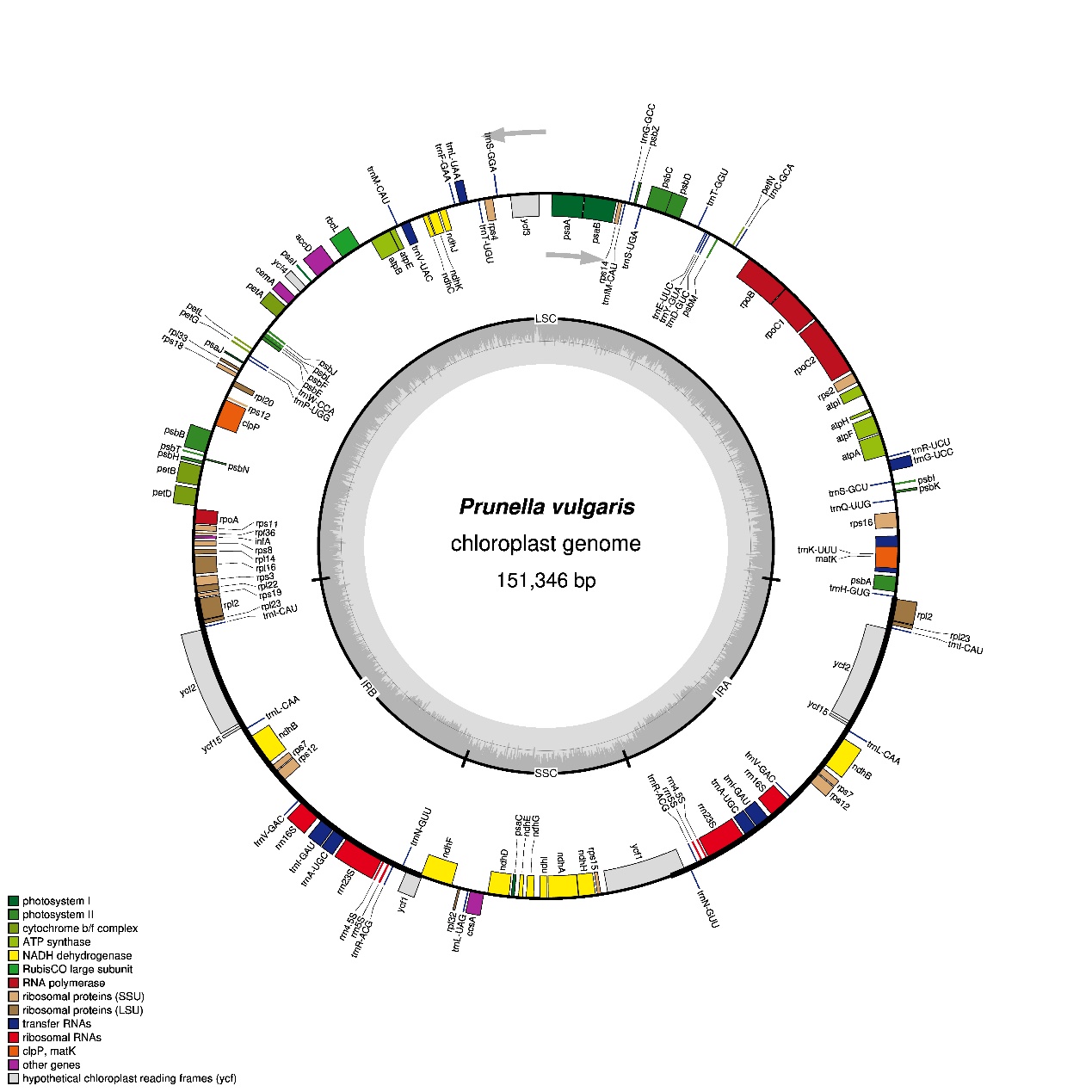
**

## Table S1. Homologous fragments of mitochondria genome and chloroplasts genome

| Number | Mitochondrial genome | % Identity | Alignment Length | Mismatches | Gap Openings | Alignment start (chloroplast genome) | Alignment end (chloroplast genome) | Alignment start (mitochondrial genome) | Alignment end (mitochondrial genome) | E-value | Bit Score | MTPT annotation |
| --- | --- | --- | --- | --- | --- | --- | --- | --- | --- | --- | --- | --- |
| 1 | Chromosome 1 | 100 | 2636 | 0 | 0 | 37975 | 40610 | 1.79E+05 | 176169 | 0.00E+00 | 4868 | Partial (*psa*B,*psa*A) |
| 2 | Chromosome 1 | 99.866 | 749 | 1 | 0 | 113117 | 113865 | 68800 | 68052 | 0 | 1378 | Partial (*ndh*D) |
| 3 | Chromosome 1 | 94.891 | 822 | 24 | 5 | 22210 | 23026 | 173158 | 173966 | 0 | 1269 | Partial (*rpo*C1) |
| 4 | Chromosome 1 | 98.96 | 673 | 3 | 2 | 116506 | 117175 | 88192 | 88863 | 0.00E+00 | 1201 | Complete (*ndh*I)，Partial (*ndh*A) |
| 5 | Chromosome 1 | 98.641 | 515 | 3 | 1 | 77173 | 77687 | 68795 | 69305 | 0.00E+00 | 909 | Partial (*rpo*A) |
| 6 | Chromosome 1 | 95.276 | 381 | 4 | 2 | 75179 | 75559 | 51356 | 51722 | 1.66E-168 | 592 | Partial (*pet*D) |
| 7 | Chromosome 1 | 99.007 | 302 | 3 | 0 | 23108 | 23409 | 173958 | 174259 | 1.69E-153 | 542 | Partial (*rpo*C1,*rpo*B) |
| 8 | Chromosome 1 | 98.707 | 232 | 2 | 1 | 63190 | 63421 | 116079 | 116309 | 4.98E-114 | 411 | Complete (*psb*J) |
| 9 | Chromosome 1 | 95.633 | 229 | 7 | 3 | 62707 | 62933 | 69507 | 69280 | 3.93E-100 | 364 | IGS(*pet*A,*psb*J) |
| 10 | Chromosome 1 | 98.611 | 144 | 2 | 0 | 84019 | 84162 | 65181 | 65038 | 2.47E-67 | 255 | Partial (*rpl*2) |
| 11 | Chromosome 1 | 98.611 | 144 | 2 | 0 | 149877 | 150020 | 65038 | 65181 | 2.47E-67 | 255 | Partial (*rpl*2) |
| 12 | Chromosome 1 | 100 | 78 | 0 | 0 | 127118 | 127195 | 7307 | 7230 | 5.58E-34 | 145 | Partial (*trn*N-GUU) |
| 13 | Chromosome 1 | 100 | 78 | 0 | 0 | 106844 | 106921 | 7230 | 7307 | 5.58E-34 | 145 | Partial (*trn*N-GUU) |
| 14 | Chromosome 1 | 93.75 | 80 | 5 | 0 | 51761 | 51840 | 137365 | 137444 | 9.4E-27 | 121 | Complete (*trn*M-CAU) |
| 15 | Chromosome 1 | 97.059 | 68 | 1 | 1 | 34617 | 34684 | 61289 | 61223 | 1.57E-24 | 113 | IGS(*psb*C,*trn*S-UGA) |
| 16 | Chromosome 1 | 98.387 | 62 | 1 | 0 | 101978 | 102039 | 43695 | 43634 | 2.03E-23 | 110 | Partial (*trn*A-UGC) |
| 17 | Chromosome 1 | 98.387 | 62 | 1 | 0 | 132000 | 132061 | 43634 | 43695 | 2.03E-23 | 110 | Partial (*trn*A-UGC) |
| 18 | Chromosome 1 | 100 | 29 | 0 | 0 | 53441 | 53469 | 173138 | 173166 | 0.000000967 | 54.7 | Partial (*atp*B) |
| 19 | Chromosome 2 | 99.176 | 3276 | 6 | 1 | 145534 | 148809 | 75786 | 72532 | 0 | 5880 | Partial (*ycf*2) |
| 20 | Chromosome 2 | 99.176 | 3276 | 6 | 1 | 85230 | 88505 | 72532 | 75786 | 0 | 5880 | Partial (*ycf*2) |
| 21 | Chromosome 2 | 99.963 | 2692 | 1 | 0 | 138455 | 141146 | 80856 | 78165 | 0 | 4966 | Complete (*ndh*B)，Partial (*rps*7) |
| 22 | Chromosome 2 | 99.963 | 2692 | 1 | 0 | 92893 | 95584 | 78165 | 80856 | 0 | 4966 | Complete (*ndh*B)，Partial (*rps*7) |
| 23 | Chromosome 2 | 100 | 2026 | 0 | 0 | 63476 | 65501 | 112247 | 114272 | 0 | 3742 | Complete (*psb*L,*psb*F,*psb*E,*pet*L,*pet*G,*trn*W-CCA) |
| 24 | Chromosome 2 | 97.256 | 2041 | 29 | 6 | 43841 | 45875 | 63848 | 65867 | 0 | 3434 | Complete (*trn*S-GGA,*rps*4) |
| 25 | Chromosome 2 | 100 | 1156 | 0 | 0 | 144121 | 145276 | 76926 | 75771 | 0 | 2135 | Partial (*ycf*2) |
| 26 | Chromosome 2 | 100 | 1156 | 0 | 0 | 88763 | 89918 | 75771 | 76926 | 0 | 2135 | Partial (*ycf*2) |
| 27 | Chromosome 2 | 100 | 736 | 0 | 0 | 141861 | 142596 | 78174 | 77439 | 0 | 1360 | Complete (*ycf*15)，Partial (*ycf*2) |
| 28 | Chromosome 2 | 100 | 736 | 0 | 0 | 91443 | 92178 | 77439 | 78174 | 0 | 1360 | Complete (*ycf*15)，Partial (*ycf*2) |
| 29 | Chromosome 2 | 98.336 | 541 | 1 | 1 | 143449 | 143989 | 77449 | 76917 | 0 | 942 | Partial (*ycf*2) |
| 30 | Chromosome 2 | 98.336 | 541 | 1 | 1 | 90050 | 90590 | 76917 | 77449 | 0 | 942 | Partial (*ycf*2) |
| 31 | Chromosome 2 | 95.614 | 228 | 5 | 1 | 41081 | 41308 | 16301 | 16523 | 3.17E-99 | 361 | IGS(*psa*A,*ycf*3) |
| 32 | Chromosome 2 | 93.388 | 242 | 7 | 1 | 65529 | 65770 | 15 | 247 | 6.85E-96 | 350 | Complete (*trn*P-UGG) |
| 33 | Chromosome 2 | 95.098 | 102 | 5 | 0 | 29626 | 29727 | 41040 | 40939 | 3.45E-39 | 161 | Complete (*trn*D-GUC) |
| 34 | Chromosome 2 | 97.531 | 81 | 2 | 0 | 8 | 88 | 61826 | 61746 | 1.62E-32 | 139 | Complete (*trn*H-GUG) |
| 35 | Chromosome 2 | 100 | 60 | 0 | 0 | 117478 | 117537 | 4493 | 4434 | 3.52E-24 | 111 | Partial (*ndh*A) |
| 36 | Chromosome 2 | 100 | 29 | 0 | 0 | 7903 | 7931 | 64724 | 64696 | 0.000000602 | 54.7 | Partial (*trn*S-GCU) |

## Table S2. The information of 22 species constituting the phylogenetic tree.

| **Lamiaceae** | **Prunella vulgaris** |  | |
| --- | --- | --- | --- |
|  | Salvia miltiorrhiza mitochondrion, complete genome | | NC_023209.1 |
|  | Rotheca serrata mitochondrion, complete genome | | NC_049064.1 |
|  | Pogostemon heyneanus mitochondrion, complete genome | | MK728874.1 |
|  | Scutellaria tsinyunensis mitochondrion, complete genome | | MW553042.1 |
|  | Scutellaria barbata mitochondrion, complete genome | | NC_065025.1 |
|  | Scutellaria franchetiana mitochondrion, complete genome | | NC_065026.1 |
|  | Ajuga reptans mitochondrion, complete genome | | NC_023103.1 |
|  | Ajuga ciliata mitochondrion, complete sequence | | MT075725_6.1 |
|  | Vitex trifolia mitochondrion, complete genome | | NC_065806.1 |
| **Orobanchaceae** | Castilleja paramensis mitochondrion, complete genome | | NC_031806.1 |
|  | Aeginetia indica mitochondrion, complete genome | | MW851294.1 |
|  | Rehmannia glutinosa mitochondrion, complete genome | | OM397952.1 |
|  | Christisonia kwangtungensis mitochondrion, complete sequence | | OM219025_7.1 |
| **Plantaginaceae** | Aragoa cleefii mitochondrion, complete genome | | OK514182.1 |
|  | Aragoa abietina mitochondrion, complete genome | | OK514181.1 |
| **Gesneriaceae** | Boea hygrometrica mitochondrion, complete genome | | NC_016741.1 |
|  | Haberlea rhodopensis mitochondrion, complete genome | | MH757117.1 |
| **Lentibulariaceae** | Utricularia reniformis mitochondrion, complete genome | | NC_034982.1 |
|  | Genlisea tuberosa voucher VFOM2001 mitochondrion, complete genome | | OK274069.1 |
| **Oleaceae** | Hesperelaea palmeri voucher E. Palmer 81 (MO) mitochondrion, complete genome | | NC_031323.1 |
|  | Osmanthus fragrans mitochondrion, complete sequence | | NC_060346.1 |

## Table S3. Relative synonymous codon usage for each amino acid pair of codons in the mitochondrial genome of *Prunella vulgaris.*

| **Amino** | **Codon 1** | **Codon 2** | **Codon 3** | **Codon 4** | **Codon 5** | **Codon 6** |
| --- | --- | --- | --- | --- | --- | --- |
|  | **RSCU** | **RSCU** | **RSCU** | **RSCU** | **RSCU** | **RSCU** |
| Ala | GCU | GCC | GCA | GCG |  |  |
|  | 1.61 | 0.96 | 0.94 | 0.49 |  |  |
| Arg | CGA | AGA | CGU | AGG | CGG | CGC |
|  | 1.32 | 1.28 | 1.25 | 0.76 | 0.76 | 0.62 |
| Asn | AAU | AAC |  |  |  |  |
|  | 1.42 | 0.58 |  |  |  |  |
| Asp | GAU | GAC |  |  |  |  |
|  | 1.36 | 0.64 |  |  |  |  |
| Cys | UGU | UGC |  |  |  |  |
|  | 1.25 | 0.75 |  |  |  |  |
| End | UAA | UGA | UAG |  |  |  |
|  | 1.55 | 0.83 | 0.62 |  |  |  |
| Gln | CAA | CAG |  |  |  |  |
|  | 1.52 | 0.48 |  |  |  |  |
| Glu | GAA | GAG |  |  |  |  |
|  | 1.42 | 0.58 |  |  |  |  |
| Gly | GGA | GGU | GGG | GGC |  |  |
|  | 1.45 | 1.31 | 0.71 | 0.53 |  |  |
| His | CAU | CAC |  |  |  |  |
|  | 1.52 | 0.48 |  |  |  |  |
| Ile | AUU | AUC | AUA |  |  |  |
|  | 1.37 | 0.85 | 0.79 |  |  |  |
| Leu | UUA | CUU | UUG | CUA | CUC | CUG |
|  | 1.58 | 1.29 | 1.13 | 0.94 | 0.61 | 0.46 |
| Lys | AAA | AAG |  |  |  |  |
|  | 1.19 | 0.81 |  |  |  |  |
| Met | AUG |  |  |  |  |  |
|  | 1 |  |  |  |  |  |
| Phe | UUU | UUC |  |  |  |  |
|  | 1.18 | 0.82 |  |  |  |  |
| Pro | CCU | CCA | CCC | CCG |  |  |
|  | 1.5 | 1.09 | 0.83 | 0.57 |  |  |
| Ser | UCU | UCA | AGU | UCC | UCG | AGC |
|  | 1.39 | 1.18 | 1.05 | 0.95 | 0.81 | 0.63 |
| Thr | ACU | ACC | ACA | ACG |  |  |
|  | 1.4 | 1.05 | 0.97 | 0.58 |  |  |
| Trp | UGG |  |  |  |  |  |
|  | 1 |  |  |  |  |  |
| Tyr | UAU | UAC |  |  |  |  |
|  | 1.51 | 0.49 |  |  |  |  |
| Val | GUU | GUA | GUG | GUC |  |  |
|  | 1.26 | 1.25 | 0.8 | 0.69 |  |  |
